# The Effects of a Campus Forest-Walking Program on Undergraduate and Graduate Students’ Physical and Psychological Health

**DOI:** 10.3390/ijerph14070728

**Published:** 2017-07-05

**Authors:** Kyung-Sook Bang, Insook Lee, Sungjae Kim, Chun Soo Lim, Hee-Kyung Joh, Bum-Jin Park, Min Kyung Song

**Affiliations:** 1 College of Nursing, The Research Institute of Nursing Science, Seoul National University, Seoul 03080, Korea; ksbang@snu.ac.kr (K.-S.B.); lisook@snu.ac.kr (I.L.); sungjae@snu.ac.kr (S.K.); 2 Department of Internal Medicine, Seoul National University College of Medicine, Seoul 03080, Korea; cslimjy@snu.ac.kr; 3 Department of Medicine, Seoul National University College of Medicine, Seoul 03080, Korea; hkjoh@snu.ac.kr; 4 Department of Family Medicine, Seoul National University Health Service Center, Seoul 08826, Korea; 5 Department of Family Medicine, Seoul National University Hospital, Seoul 03080, Korea; 6 Department of Environment and Forest Resources, College of Agriculture and Life Sciences, Chungnam National University, Daejeon 34134, Korea; bjpark@cnu.ac.kr; 7 College of Nursing, Seoul National University, Seoul 03080, Korea

**Keywords:** forests, walking, health promotion, body composition, depression, college students

## Abstract

We conducted a campus forest-walking program targeting university and graduate students during their lunchtime and examined the physical and psychological effects of the program. We utilized a quasi-experimental design with a control group and a pretest–posttest design. Forty-seven men (M = 25.5 ± 3.8 years) and 52 women (M = 23.3 ± 4.3 years) volunteered to participate (experimental group *n* = 51, control group *n* = 48). The intervention group participated in campus forest-walking program once a week for six weeks; they were also asked to walk once a week additionally on an individual basis. Additionally, participants received one lecture on stress management. Post-tests were conducted both just after the program ended and three months after. A chi-square test, *t*-test, and repeated measures analysis of variance were used to evaluate the effects of the program. Health promoting behaviors (*F* = 7.27, *p* = 0.001, ES = 0.27) and parasympathetic nerve activity (*F* = 3.69, *p* = 0.027, ES = 0.20) significantly increased and depression (*F* = 3.15, *p* = 0.045, ES = 0.18) significantly decreased in the experimental group after the intervention compared to the control group. In conclusion, using the campus walking program to target students during their lunchtime is an efficient strategy to promote their physical and psychological health.

## 1. Introduction

One of the most important public health problems today is individuals’ lack of physical activity [1]. The World Health Organization has recommended that adults aged 18–64 years should do at least 150 min of moderate-intensity aerobic physical activity or at least 75 min of vigorous-intensity aerobic physical activity or an equivalent combination of moderate- and vigorous-intensity activity throughout the week [2]. The benefits of physical activity include lowering the rates of all-cause mortality, coronary heart disease, high blood pressure, stroke, type-2 diabetes, metabolic syndrome, and depression [3].

The college years are a time of transition from adolescence to adulthood [4], and usually involve students obtaining independence from their parents. The college years are also a crucial time for health promotion, disease prevention, and forming lifestyle patterns for later life [5,6]. College students are hopefully of optimal health and well-being; however, they are exposed to several health risk factors including irregular sleep patterns, personal relationship changes, overdrinking, and academic pressures [7], and they experience a large amount of stress, anxiety, and depression [8]. According to repeated previous studies, approximately 50% of college students experience significant levels of stress, anxiety, or depression, or both [9]. A study of 5245 Chinese university students found that older students were sensitive to depression compared to younger students because older students face more stressful events, such as employment, economic, graduation, and marriage pressures [10]. Moreover, students with mental health problems show poor relationships with other students, low grade averages, low rates of graduation, and a high incidence of suicide or self-harm behavior [11,12]. In addition, many students are at an elevated risk of metabolic syndrome caused by a lack of exercise and excessive drinking [13]. Therefore, the mental health of college students including stress, anxiety, and depression and their exercise habits are significant issues.

Although there has been a growing interest in health promotion, it is not easy for university students to maintain an appropriate level of physical activity because their academic responsibilities are often recognized as being more important. Lunchtime is a common ‘break’ for college students, and partaking in physical activity during lunchtime may help increase overall activity levels [14].

Walking is a universal, convenient, familiar, and free-form physical activity that has diverse health benefits and a low injury risk [15]. Recently, more people have become interested in health habits such as maintaining a healthy diet, exercising, and partaking in outdoor activities. Walking improves several markers of cardiovascular risk, including aerobic capacity, systolic and diastolic blood pressure, waist circumference, body fat percentage, and body mass index [16].

Walking in the forest has especially been found to have more positive effects on physical and psychological health than walking in the city [17,18]. The natural environment is increasingly recognized as an effective counter to urban stress [19]. Higher accessibility to parks or forests is associated with higher happiness and a better mood as well as less stress, anger, and depression [20]. Many studies have examined the effect of this ‘forest therapy’ on human health. They have revealed many beneficial effects such as lowering blood pressure in hypertension patients, decreasing sympathetic nerve activity, enhancing parasympathetic nerve activity, and the activation of natural killer cells [21,22,23,24]. Forest walking has also been shown to significantly increase people’s positive emotions and decrease their negative emotions compared with activities in urban areas [25,26].

This study utilized the Information–Motivation–Behavioral skills (IMB) model to design the intervention to promote physical and mental health. The IMB model has received considerable attention because it not only provides a relatively simple explanation for complex health behaviors, but also identifies constructs (including information, motivation, and behavioral skills) that are needed for successful self-management or adherence [27]. The IMB model, proposed by Fisher and Fisher to explain human immunodeficiency virus (HIV) related behaviors, recognizes three constructs: information, motivation, and behavioral skills as specific individual determinants of behavior and behavioral change [28,29]. According to this theory, changing not only the physical environment, but also attitudes and behaviors toward health are required for one’s health. In addition, it is necessary to provide information based on scientific data and motivation to increase interest in health. Using this IMB model as the conceptual framework of the campus forest-walking program for students, it is possible to develop systematic interventions to promote health.

To date, however, studies on the effects of forest walking for college students who suffer from elevated levels of stress are limited. Additionally, few studies have used robust data collection methods to measure the impact of on-campus interventions on college students’ physical activity levels, mental health, and health biomarkers. Therefore, this study identified the effects of an on-campus, forest-walking program for college students and included both objective and subjective measures such as physical activity level; health promoting behavior; and physical, physiological, and psychological biomarkers of health [28,29]. The specific research questions for the study were as follows: Does the six-week campus walk program reduce depression and improve relaxation among college and graduate students? Does this program improve the health promotion behavior and increase physical activity of the students? We will test the following four hypotheses in this study:

1. The experimental group participating in the program will display a higher score on health promoting behaviors than the control group will.
2. The experimental group will display a lower depression score than the control group will.
3. The experimental group will display more parasympathetic nerve activity than the control group will.
4. The physical health (i.e., bone density, blood lipid profile, and body mass index) of the experimental group will be better than that of the control group.

## 2. Methods

### 2.1. Study Design and Participants

This was a quasi-experimental study with a control group and a pretest–posttest design. The study occurred from September 2014 to February 2015 (from fall to the end of winter). For the intervention group, the campus forest-walking program was provided for six weeks, and the effects of the program regarding participants’ physical and mental health were analyzed. The pre-test took place about a week before the intervention started, the post-test was conducted one week after the final program, and the follow-up test was conducted after another three months.

**Figure 1:**
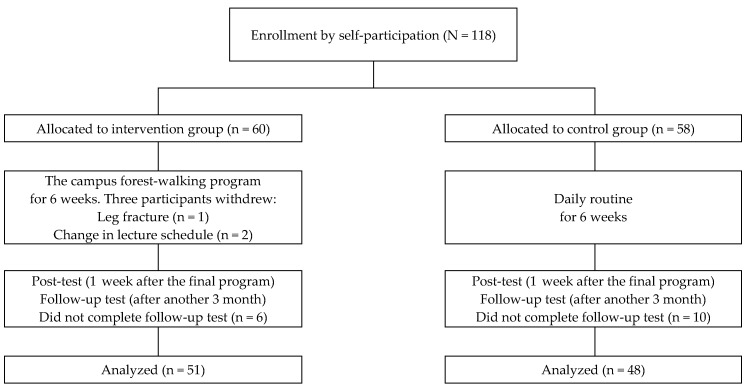
Recruitment of participants.

Participants comprised graduate and undergraduate students from one university in Seoul, South Korea. Participants were recruited by posting notices using the university homepage and sending an e-mail to all students from the university health service center. Students who had medical contraindications to exercise by self-report (e.g., asthma, painful osteoarthritis, or heart conditions) were excluded [30]. One-hundred and eighteen students voluntarily participated in this study. To increase motivation, group assignment (experimental or control) was made per participants’ preference (Figure 1).

This study was conducted in accordance with the Declaration of Helsinki and was approved by the Ethics Committee of the Institutional Review Board at Seoul National University in Seoul, Republic of Korea (IRB No. 1409/001-001). Before beginning the study, a full explanation about the research purpose, the experimental procedure, and all measurement indices were provided to participants. Those who agreed to participate were selected as study participants who provided full written consent for taking part in the program (experimental group *n* = 60, control group *n* = 58). Three of the participants withdrew their participation during the study period. Sixteen participants did not complete the follow-up test because of employment, birth, a heavy schedule, or refusal.

### 2.2. Intervention

The six-week campus forest-walking program was conducted once a week during lunch. The university campus where the program was implemented is located just northwest of the mountain Gwanack and has many different trees. In addition, there are forest roads and trails near the campus.

**Figure 2:**
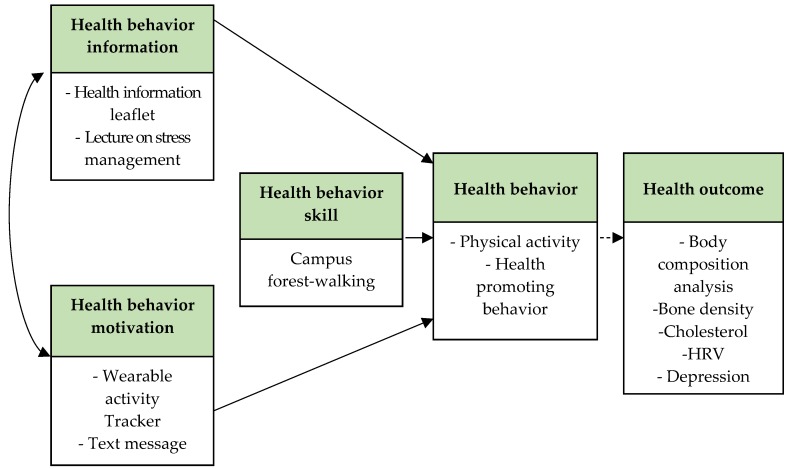
The conceptual model of the campus forest-walking program.

The information consisted of lectures on stress management and providing leaflets related to mental and physical health. We sent a text message to promote voluntarily walking once a week. Health leaflets about the effects of forest therapy; the correct walking method; and self-efficacy for walking, stress management, and depression management were provided at the first session of the program. In addition, participants in the experimental group were provided with a wearable activity tracker, Fitbit Zip^®^ (Fitbit Inc., San Francisco, CA, USA), which allowed them to self-monitor their physical activity (Figure 2).

The intervention was provided from September to October 2014, once a week during lunchtime or for one hour on Wednesdays at 4 p.m., depending on participants’ preferred schedule. Participants in the experimental group walked together in the campus forest at a relaxed pace for about 40 min, with a 10 min rest during the walk, and had a light meal (e.g., sandwich) during the walk.

During the intervention period, we also provided one lecture on stress management. The average group size was 10 (not more than 15). They were also encouraged through a text message to additionally walk at least once a week at their leisure. The control group did not receive leaflets, lectures, or a wearable activity tracker and were asked to follow their routine activity during the study period. The intervention group and control group were asked to participate in pre-, post-, and follow-up tests.

### 2.3. Measurements

Questionnaires were administered to investigate demographic data, health promoting behavior, physical activity level, and depression. Demographic data included age, sex, and academic year.

Health promoting behavior was assessed using the Korean translation of the Health‑Promoting Lifestyle Profile II (HPLP‑II) [31]. The HPLP‑II is a 50‑item measure; answers are provided using a four‑point Likert scale based on Pender’s health promotion model, which contains six subscales: responsibility for health, physical activity, healthy nutrition, social relations, stress management, and spiritual growth. The total scores of the HPLP-II range from 50 to 200, with a higher score indicating a better health-promoting lifestyle. The Cronbach’s α for the total instrument was 0.92 and the six subscales ranged from 0.65 to 0.82 [26]. In this study, the Cronbach’s α = 0.91 and the six subscales ranged from 0.70 to 0.87.

Physical activity was measured using the International Physical Activity Questionnaire-Short Form, which provides a measure of total physical activity accrued through work and leisure in all settings combined. Participants were asked about frequency and duration of exercise by indicating days and time spent doing vigorous and moderate activities, walking, and sitting in the last seven days. These activity categories may be treated separately to obtain the specific activity patterns or multiplied by their estimated value in Metabolic Equivalent of Tasks (MET) and summed to gain an overall estimate of physical activity in a week.

- Walking MET-minutes/week = 3.3 × walking minutes × walking days
- Moderate MET-minutes/week = 4.0 × moderate-intensity activity minutes × moderate days
- Vigorous MET-minutes/week = 8.0 × vigorous-intensity activity minutes × vigorous-intensity days.

Total physical activity MET-min/week, which can be calculated as the sum of walking + normal + active MET-minutes/week scores [32], was used for the analysis.

Depression level was measured using the Beck Depression Inventory. It is composed of 21 items and is answered using a four-point (0–3) Likert scale. The total sum of the item scores ranged from 0 to 63, with higher total scores indicating more severe depressive symptoms.

Height and weight were measured with an automatic stadiometer (BSM 370, Inbody Co., Ltd., Seoul, Korea). Body composition was assessed including amount of body fat, body fat percentage, body mass index, skeletal muscle mass, and amount of muscle by body composition analyzer (Inbody 570, Inbody Co.). After 10 min of resting, blood pressure was measured. Bone density was measured at the right calcaneus bone using broadband ultrasound attenuation (Sonost 3000, OsteoSys Co., Ltd., Seoul, Korea). In general, bone density indicates the value with a *t*-score. “−1 and above” the *t*-score is normal; “−2.5 to −1” means osteopenia, a condition where the bone density is below normal, which may lead to osteoporosis; and “−2.5 or below” means osteoporosis [33].

Heart Rate Variability (HRV) was measured using a portable electrocardiograph (LXC3203, LAXTHA Inc., Daejeon, Korea). HRV data were obtained at various frequency bands using an HRV software tool (TeleScan, LAXTHA Inc., Daejeon, Korea). After 10 min of resting, HRV was measured. A heart rate monitor using unipolar limb lead electrocardiogram (ECG) recorder was used to collect continuous RR intervals, which were stored on computer for later analysis. It took approximately 5 min to measure this. Among commonly used HRV indices, the low-frequency (LF: 0.04–0.15 Hz) power of the HRV Fourier spectrum has been presumed to reflect some aspects of cardiac sympathetic modulation, and the ratio of LF power to high-frequency (HF: 0.15–0.40 Hz) power (LF/HF ratio) indicates the sympathovagal balance [34]. Parasympathetic nerve activity is high when participants are calm. It is widely used to measure the mediation effect of mind and body relaxation [35].

Blood samples were taken to determine fasting serum total cholesterol, low-density lipoprotein cholesterol, high-density lipoprotein cholesterol, and triglyceride. Participants were asked not to eat or drink for 12 h before testing. One laboratory determined all blood biochemistry parameters (Green Cross Laboratories, Gyeonggi-do, Korea).

### 2.4. Statistics

Statistical analyses were performed using a Windows-Based Statistical Package version 22.0 software (SPSS, Chicago, IL, USA). Descriptive statistics comprised mean, standard deviation, frequency, and percentage to present demographic information and outcome variables. An independent *t*-test and χ^2^ test were conducted to test homogeneity at baseline between the experimental and control groups. For this study, a repeated-measures analysis of variance (rmANOVA) was used to analyze the effects of intervention directly after the program and three months later. All statistical tests were two-tailed and a *p*-value < 0.05 was considered statistically significant.

## 3. Results

### 3.1. Homogeneity Test of the Experimental and Control Group

Participants’ mean age was 24.3 ± 4.19 years. A homogeneity test of the general characteristics between the experimental and control groups in pretest showed no significant difference (Table 1).

### 3.2. Effects of the Intervention on Outcome Measures

To analyze the effect of the intervention, a rmANOVA and Mauchly’s test of sphericity were used. Mauchly’s test for sphericity, a test of homogeneity of variance, tests the null hypothesis (*p* > 0.05) that the differences between variances comparing all possible pairs of groups are equal. In those cases, when the null hypothesis is not true (e.g., high-density lipoprotein (HDL), low-density lipoprotein (LDL), Bone density, body mass index (BMI), low frequency/high frequency (LF/HF) ratio), we used a multivariate analysis of variance (MANOVA) [36].

**Figure 3:**
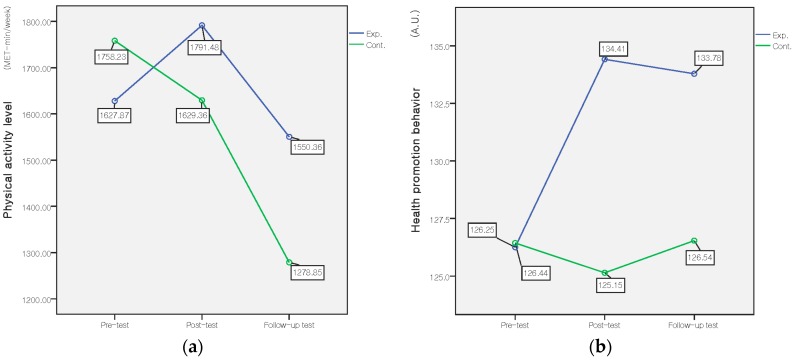
Comparison of the mean physical activity level (**A**) and health promotion behavior score (**B**) between the experimental and control groups at pre-, post-, and follow-up test. Exp.: experimental group; Cont.: control group; A.U.: arbitrary units.

The interaction effect of group (experimental and control) and time (pre-intervention, post-intervention, and three-months after the intervention) were tested. Mean scores of physical activity level and health promoting behavior for experimental and control groups are presented in Table 2 and Figure 3. The results of the repeated measure ANOVA showed that there was a significant group × time effect for the health promoting behavior (*F* = 7.27, *p* = 0.001, ES = 0.27). Among the six subscales of health promoting behavior, there were significant group × time effects for physical activity (*F* = 5.91, *p* = 0.003, ES = 0.25), healthy nutrition (*F* = 3.64, *p* = 0.028, ES = 0.19), stress management (*F* = 3.32, *p* = 0.038, ES = 0.18), and spiritual growth (*F* = 3.14, *p* = 0.045, ES = 0.18). There was a significant difference in health promoting behavior in the time by group interaction effect; however, no interaction effect was found for physical activity level.

**Figure 4:**
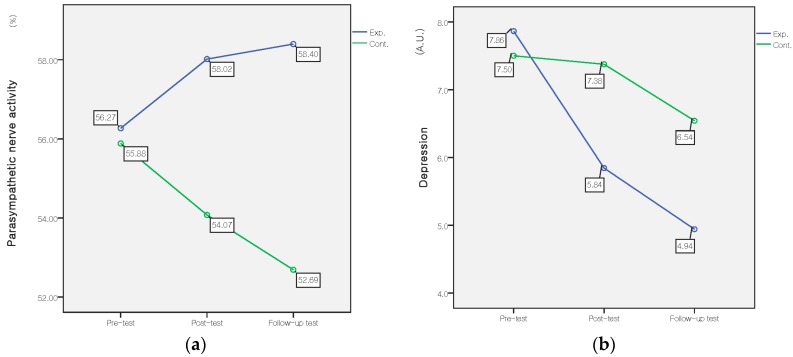
Comparison of the parasympathetic nerve activity (**A**) and mean depression score (**B**) between the experimental and control groups in pre-, post-, and follow-up tests.

For physiological measures, only the body fat percentage showed a significant interaction effect (*F* = 3.41, *p* = 0.035, ES = 0.19). Concerning HRV, the interaction effect for parasympathetic nerve activity was significant (*F* = 3.69, *p* = 0.027, ES = 0.20). For psychological subjective measures, depression showed a significant interaction effect of time by group (*F* = 3.15, *p* = 0.045, ES = 0.18), (Table 3 and Table 4, Figure 4).

## 4. Discussion

We evaluated the effects of an on-campus, forest-walking program on the physical and psychological health of undergraduate and graduate students. Pre-, post-, and follow-up variables were measured to compare an experimental group and a control group.

Participants in the intervention group had a higher score on health promoting behavior than the control group did, especially physical activity, health nutrition, stress management, and spiritual growth scores among the six subscales. This is consistent with the effect of resistance band exercise (low intensity exercise) among nursing students [37]. Although the campus forest-walking program was a low intensity activity for six weeks, it provided the opportunity to be motivated by periodic group walking, self-monitoring, and concentrating on health. It is meaningful that this intervention positively affected the health-promotion behavior of participants—including a healthy diet, physical activity, stress management, and spiritual growth—because this will subsequently lead to better lifestyle. The pleasant experience of forest walking increased participants’ interest in health care, and the stress management education may have influenced this positive effect.

In this study, the campus forest-walking program had no significant effect on physical activity level. Moreover, physical activity level decreased in both groups at the three-month follow-up, and significant differences were only shown for the time variable. The intervention program was provided in the autumn (September to November 2014), and the follow-up test was conducted at the end of winter (February 2015). The wintry weather could affect the level of physical activity for both groups. Therefore, the decrease in PA may have been affected by the timing of the program. Moreover, because winter has fewer sunlight hours and disadvantageous weather conditions, physical activity may be less convenient than it is in the summer [38]. Walking programs are known to be effective in promoting physical activity. Therefore, we can consider various strategies for future research: interventions tailored to people’s needs; targeting the most sedentary or the most motivated to change; and intervening at either the individual, household, or group level [39].

The effectiveness on psychological health, which was shown with higher levels of parasympathetic nerve activation and decreased depression, is the most noteworthy aspect of the forest-walking intervention. The results of increased parasympathetic activity in the experimental group are consistent with previous studies of physiological responses to the forest environment, suggesting that walking in forests may provide health benefits of relaxation [35,40,41]. Numerous forest-walking studies have shown the effects on relieving negative emotions such as anxiety and depression [17,18,42,43,44]. Walking in the forest has also been shown to be more effective on mental health than walking in the city due to less environmental stress [45]. Young adults who walked for 50 min in nature after a stressful situation showed reduced stress and anger and increased positive affect [46]. Our results add evidence to the known positive effects of forest walking in increasing mental relaxation and reducing depression. Therefore, forest walking could be a simple, accessible and low-cost strategy to improve physical and mental health.

We acknowledge the limitations of this study. The limitations include the lack of randomization, which may lead to selection bias. In addition, participants chose to take part either in the intervention or control groups: attitudes, motivation, and personal factors might have caused a selection bias. Participants were highly motivated and were willing to volunteer to be in the experimental group. People who are highly motivated to change their behavior may be more inclined to take part in an intervention and more likely to benefit from the intervention. Although the two groups did not seem to differ significantly at baseline in relation to the measures, we did not control for personal orientation or motivational processes; therefore, the findings may not be directly generalizable to all college students. In addition, the intervention group was required to exercise on their own once a week. Although the physical activity level was measured with a Fitbit, the Fitbit data were not evaluated. Additionally, we did not know if within the intervention group some students engaged in self-walking more than others. Social factors related to performing an activity with others could have played a role. Moreover, small intervention groups were selected according to individuals’ schedules. While walking, participants could see and ‘feel’ the forest without talking to each other or looking at a cell phone; however, we cannot rule out the possibility that the nature of the program and meeting new people could have affected the outcome. Finally, because the walking program was offered to participants once per week, the frequency may be different from other intervention studies, which makes comparisons between studies difficult.

Despite these limitations, our study has several notable strengths including the use of the campus forest, which is easily accessible to students, and performing the program at lunch time, which is convenient. In addition, the physiological and psychological results of this experiment provide evidence for the physiological and psychological benefits of campus forests. Walking in the campus forest reduced depression by increasing parasympathetic activity. In addition, we examined participants at a three-month follow-up and included multiple outcome variables such as physiological measures and self-reported measures.

## 5. Conclusions

This study showed that a six-week, on-campus, forest-walking program produced beneficial changes in the physical and psychological health of undergraduate and graduate students. The intervention significantly increased participants’ health promoting behavior and parasympathetic nerve activity compared to the control group, as well as significantly decreasing depression.

Further research is needed to examine changes in exercise frequency and duration. A study with a larger sample size is required to address the generalizability of the results. The random assignment of participants and manipulating potential confounding factors of the intervention are also needed. However, this study suggests a campus forest-walking program as a strategy to promote students’ physical and mental health, thereby contributing to the health promotion and a healthy culture among university and graduate students. Therefore, students should be encouraged to take advantage of the forest during their lunch hour as it may positively affect their quality of life.

## Figures and Tables

**Table 1:** Homogeneity test of participants’ general characteristics and outcome variables during the pre-test (*N* = 99).

Characteristics/Variables	Categories	Exp. (*n* = 51) *n* (%) M ± SD	Cont. (*n* = 48) *n* (%) M ± SD	*x*^2^ or *t*	*p*
Age (years)		24.8 ± 4.66	23.8 ± 3.60	1.29	0.201
Sex	Male	26 (51.0)	21 (43.8)	0.518	0.548
	Female	25 (49.0)	27 (56.3)		
College standing	Undergraduate	23 (45.1)	23 (47.9)	0.079	0.842
	Graduate	28 (54.9)	25 (52.1)		
Blood pressure (mmHg)	Systolic BP	111.55 ± 11.45	107.85 ± 11.87	1.58	0.118
	Diastolic BP	68.47 ± 9.64	67.54 ± 7.83	0.52	0.601
Blood cholesterol (mg/dL)	Cholesterol, total	176.59 ± 31.79	176.25 ± 30.73	0.05	0.957
	HDL	65.06 ± 15.20	68.58 ± 14.07	−1.20	0.235
	LDL	103.49 ± 29.01	97.75 ± 26.91	1.02	0.311
	TG	76.92 ± 42.46	71.90 ± 35.53	0.64	0.526
Bone density, *t*-score		−0.83 ± 0.92	−0.90 ± 1.05	0.33	0.743
BMI (kg/m^2^)		21.91 ± 2.87	21.40 ± 2.77	0.89	0.375
Body composition	Percent of body fat (%)	24.09 ± 6.79	23.47 ± 6.38	0.47	0.637
	Amount of muscle (kg)	44.28 ± 9.39	42.33 ± 8.89	1.06	0.290
	Skeletal muscle mass (kg)	26.03 ± 6.04	24.74 ± 5.75	1.09	0.280
Physical activity level (MET-min/week)		1627.87 ± 1620.76	1758.23 ± 1228.39	−0.45	0.655
Health promoting behavior		126.25 ± 17.80	126.44 ± 18.46	−0.05	0.960
Heart rate variability	LF/HF ratio	2.03 ± 1.59	2.21 ± 2.15	−0.47	0.636
	Parasympathetic nerve activity (%)	56.27 ± 9.34	55.88 ± 10.29	0.20	0.846
Depression		7.86 ± 5.40	7.50 ± 5.34	0.34	0.738

Exp.: experimental group; Cont.: control group; M: mean; SD: standard deviation; BP: blood pressure; LF: low-frequency; HF: high-frequency; HDL: high-density lipoprotein; LDL: low-density lipoprotein; TG: triglyceride; BMI: body mass index; MET: metabolic equivalent of tasks.

**Table 2:** Group comparisons of health promotion behavior and physical activity at pre-, post-, and follow-up test.

Variables		Time	Exp.	Cont.	Source	*F*	*p*	ES
			M ± SD					
Physical activity level (MET-min/week)		Pre-test	1627.87 ± 1620.76	1758.23 ± 1228.39	G	0.19	0.661	0.04
		Post-test	1791.48 ± 1434.68	1629.36 ± 1270.70	T	3.01	0.047	0.18
		F/U test	1550.36 ± 1310.35	1278.85 ± 1333.96	G*T	1.21	0.300	0.11
Health promoting behavior	Sum	Pre-test	126.26 ± 7.80	126.44 ± 18.46	G	2.58	0.112	0.16
		Post-test	134.41 ± 15.87	125.15 ± 20.12	T	5.19	0.006	0.23
		F/U test	133.78 ± 18.15	126.54 ± 20.11	G*T	7.27	0.001	0.27
	Responsibility for health	Pre-test	17.61 ± 4.41	16.85 ± 4.18	G	2.32	0.131	0.15
		Post-test	18.96 ± 4.48	17.33 ± 4.23	T	6.40	0.002	0.26
		F/U test	18.88 ± 4.57	17.63 ± 4.20	G*T	0.97	0.380	0.33
	Physical activity	Pre-test	18.47 ± 5.54	19.19 ± 6.11	G	1.36	0.247	0.12
		Post-test	21.00 ± 4.76	18.94 ± 6.12	T	3.05	0.050	0.18
		F/U test	20.65 ± 5.09	18.54 ± 5.95	G*T	5.91	0.003	0.25
	Healthy nutrition	Pre-test	21.59 ± 4.75	21.94 ± 5.23	G	0.59	0.443	0.08
		Post-test	22.31 ± 4.18	20.96 ± 5.59	T	0.26	0.775	0.06
		F/U test	22.45 ± 4.58	21.31 ± 5.71	G*T	3.64	0.028	0.19
	Social relations	Pre-test	23.80 ± 4.54	24.06 ± 3.86	G	0.10	0.754	0.03
		Post-test	24.75 ± 4.02	24.02 ± 3.83	T	1.27	0.282	0.11
		F/U test	24.35 ± 3.88	24.13 ± 3.87	G*T	1.46	0.235	0.12
	Stress management	Pre-test	18.43 ± 3.89	18.00 ± 3.50	G	4.18	0.044	0.21
		Post-test	20.18 ± 2.96	18.13 ± 4.08	T	12.73	0.000	0.36
		F/U test	20.49 ± 3.51	19.11 ± 3.57	G*T	3.32	0.038	0.18
	Spiritual growth	Pre-test	26.35 ± 4.58	26.50 ± 4.89	G	0.72	0.398	0.08
		Post-test	27.22 ± 4.29	25.85 ± 4.93	T	0.06	0.941	0.03
		F/U test	26.96 ± 4.49	25.98 ± 4.68	G*T	3.14	0.45	0.18

G: group; T: time; F/U: follow up; ES: effect size.

**Table 3:** Group comparisons of physiological health at pre-, post-, and follow-up test.

Variables		Time	Exp.	Cont.	Source	*F*	*p*	ES
			M ± SD					
BP (mmHg)	Systolic	Pre-test	111.55 ± 11.45	107.85 ± 11.87	Group	0.92	0.339	0.10
		Post-test	112.02 ± 11.92	110.56 ± 11.62	Time	7.83	0.001	0.28
		F/U test	114.08 ± 12.58	113.21 ± 11.74	G*T	1.11	0.333	0.11
	Diastolic	Pre-test	68.47 ± 9.64	67.54 ± 7.83	Group	0.60	0.441	0.08
		Post-test	69.29 ± 8.98	66.63 ± 8.28	Time	0.37	0.694	0.06
		F/U test	68.39 ± 8.01	68.92 ± 7.36	G*T	1.55	0.215	0.13
Cholesterol (mg/dL)	Total	Pre-test	176.59 ± 31.79	176.25 ± 30.73	Group	0.09	0.772	0.03
		Post-test	175.69 ± 28.70	177.15 ± 27.80	Time	2.01	0.137	0.14
		F/U test	177.73 ± 29.53	181.50 ± 29.82	G*T	0.63	0.535	0.08
	HDL *	Pre-test	65.06 ± 15.20	68.58 ± 14.07	Group	0.80	0.375	0.09
		Post-test	64.22 ± 14.65	65.81 ± 12.42	Time	1.86	0.161	0.20
		F/U test	64.82 ± 16.20	66.92 ± 15.63	G*T	0.46	0.635	0.10
	LDL *	Pre-test	103.49 ± 29.01	97.75 ± 26.91	Group	0.19	0.663	0.04
		Post-test	100.84 ± 26.80	99.25 ± 22.70	Time	3.97	0.022	0.29
		F/U test	103.75 ± 26.70	104.69 ± 24.51	G*T	1.51	0.225	0.18
	TG	Pre-test	76.92 ± 42.46	71.90 ± 35.53	Group	0.07	0.790	0.03
		Post-test	80.24 ± 46.40	85.60 ± 55.16	Time	3.08	0.048	0.18
		F/U test	73.59 ± 41.43	79.67 ± 45.16	G*T	1.53	0.220	0.13
Bone density, *T*-score *		Pre-test	−0.84 ± 0.92	−0.90 ± 1.05	Group	0.33	0.565	0.05
		Post-test	−0.50 ± 0.96	−0.55 ± 1.03	Time	27.04	0.000	0.75
		F/U test	−0.36 ± 0.94	−0.57 ± 0.91	G*T	1.52	0.224	0.18
BMI (kg/m^2^) *		Pre-test	21.91 ± 2.87	21.40 ± 2.77	Group	0.69	0.407	0.08
		Post-test	22.07 ± 2.91	21.47 ± 2.81	Time	2.61	0.079	0.23
		F/U test	21.99 ± 2.84	21.67 ± 3.04	G*T	1.94	0.147	0.20
Percent of body fat (%)		Pre-test	24.09 ± 6.79	23.47 ± 6.38	Group	0.41	0.524	0.06
		Post-test	24.42 ± 6.52	22.91 ± 6.96	Time	0.62	0.540	0.08
		F/U test	23.73 ± 6.58	23.33 ± 6.95	G*T	3.41	0.035	0.19

Note: * Multivariate test statistics (MANOVA), Wilks’ λ, because the assumption of spherical formation was violated.

**Table 4:** Group comparisons of psychological health at pre-, post-, and follow-up test.

Variables	Time	Exp.	Cont.	Source	F	*p*	ES
		M ± SD					
LF/HF ratio *	Pre-test	2.03 ± 1.59	2.21 ± 2.15	Group	0.363	0.548	0.06
	Post-test	1.97 ± 1.85	1.85 ± 1.27	Time	1.139	0.324	0.15
	F/U test	1.63 ± 1.27	2.07 ± 1.76	G*T	1.867	0.160	0.20
Parasympathetic nerve activity (%)	Pre-test	56.27 ± 9.34	55.88 ± 10.29	Group	4.11	0.045	0.21
	Post-test	58.02 ± 9.20	54.07 ± 10.43	Time	0.18	0.836	0.04
	F/U test	58.40 ± 8.74	52.69 ± 11.90	G*T	3.69	0.027	0.20
Depression	Pre-test	7.86 ± 5.40	7.50 ± 5.34	Group	0.92	0.340	0.10
	Post-test	5.84 ± 5.00	7.38 ± 6.02	Time	9.59	0.000	0.31
	F/U test	4.94 ± 4.82	6.54 ± 5.89	G*T	3.15	0.045	0.18

* Multivariate test statistics (MANOVA), Wilks’ λ, because the assumption of spherical formation was violated.
